# Persistent mutant oncogene specific T cells in two patients benefitting from anti-PD-1

**DOI:** 10.1186/s40425-018-0492-x

**Published:** 2019-02-11

**Authors:** Kellie N. Smith, Nicolas J. Llosa, Tricia R. Cottrell, Nicholas Siegel, Hongni Fan, Prerna Suri, Hok Yee Chan, Haidan Guo, Teniola Oke, Anas H. Awan, Franco Verde, Ludmila Danilova, Valsamo Anagnostou, Ada J. Tam, Brandon S. Luber, Bjarne R. Bartlett, Laveet K. Aulakh, John-William Sidhom, Qingfeng Zhu, Cynthia L. Sears, Leslie Cope, William H. Sharfman, Elizabeth D. Thompson, Joanne Riemer, Kristen A. Marrone, Jarushka Naidoo, Victor E. Velculescu, Patrick M. Forde, Bert Vogelstein, Kenneth W. Kinzler, Nickolas Papadopoulos, Jennifer N. Durham, Hao Wang, Dung T. Le, Sune Justesen, Janis M. Taube, Luis A. Diaz, Julie R. Brahmer, Drew M. Pardoll, Robert A. Anders, Franck Housseau

**Affiliations:** 1 Bloomberg-Kimmel Institute for Cancer Immunotherapy, Johns Hopkins University, Baltimore, MD USA; 2 Sidney Kimmel Comprehensive Cancer Center, Johns Hopkins University, Baltimore, MD USA; 3 Department of Pathology, Johns Hopkins University, Baltimore, MD USA; 4 Russell H. Morgan Department of Radiology and Radiological Science, Johns Hopkins University, Baltimore, MD USA; 5 Division of Biostatistics and Bioinformatics, Johns Hopkins University, Baltimore, MD USA; 6 The Swim Across America Laboratory, John Hopkins University, Baltimore, MD USA; 7 Ludwig Center and Howard Hughes Medical Institute, Johns Hopkins University, Baltimore, MD USA; 8 Immunitrack, Copenhagen, Denmark; 9 Department of Medicine, Division of Solid Tumor Oncology, Memorial Sloan Kettering Cancer Center, New York, NY USA; 10 Present address: B.R.B.,Bioinformatics Core, Department of Complementary & Integrative Medicine, University of Hawaii John A. Burns School of Medicine, Honolulu, HI 96813 USA

**Keywords:** Checkpoint blockade, Predictive biomarkers, Oncogene, Neoantigens, T cells

## Abstract

**Background:**

Several predictive biomarkers are currently approved or are under investigation for the selection of patients for checkpoint blockade. Tumor PD-L1 expression is used for stratification of non-small cell lung (NSCLC) patients, with tumor mutational burden (TMB) also being explored with promising results, and mismatch-repair deficiency is approved for tumor site-agnostic disease. While tumors with high PD-L1 expression, high TMB, or mismatch repair deficiency respond well to checkpoint blockade, tumors with lower PD-L1 expression, lower mutational burdens, or mismatch repair proficiency respond much less frequently.

**Case presentation:**

We studied two patients with unexpected responses to checkpoint blockade monotherapy: a patient with PD-L1-negative and low mutational burden NSCLC and one with mismatch repair proficient colorectal cancer (CRC), both of whom lack the biomarkers associated with response to checkpoint blockade, yet achieved durable clinical benefit. Both maintained T-cell responses in peripheral blood to oncogenic driver mutations – BRAF-N581I in the NSCLC and AKT1-E17K in the CRC – years after treatment initiation. Mutation-specific T cells were also found in the primary tumor and underwent dynamic perturbations in the periphery upon treatment.

**Conclusions:**

These findings suggest that T cell responses to oncogenic driver mutations may be more prevalent than previously appreciated and could be harnessed in immunotherapeutic treatment, particularly for patients who lack the traditional biomarkers associated with response. Comprehensive studies are warranted to further delineate additional predictive biomarkers and populations of patients who may benefit from checkpoint blockade.

**Electronic supplementary material:**

The online version of this article (10.1186/s40425-018-0492-x) contains supplementary material, which is available to authorized users.

## Background

Expression of the ligand for PD-1, PD-L1, by tumor cells as well as detection of intratumoral microsatellite instability (MSI-H) were approved as inclusion criteria for anti-PD1 treatment of cancer patients. Clinical response to PD-1 blockade is much more frequent in patients whose tumors have a very high nonsynonymous tumor mutational burden (TMB) and consequent neoantigen expression [1–3], likely indicating the role of T-cells specific for mutation associated neoantigens in facilitating tumor regression. For example, mismatch repair deficient colorectal cancers (MMRd CRC, also MSI-H) which typically have > 1000 mutations per exome, have an inflamed tumor microenvironment and respond well to PD-1 pathway blockade. In contrast, metastatic mismatch repair proficient CRC (MMRp mCRC, also called microsatellite stable, MSS), which are characterized by a much lower mutational burden and an uninflamed tumor microenvironment [4], rarely respond to PD-1 pathway blockade [1]. Similarly, in non-small cell lung cancer (NSCLC), high TMB cancers respond to checkpoint blockade at significantly higher frequency than those with low TMB [2, 3]. However, some patients without MMRd or high TMB cancers derive clinical benefit from PD-1 pathway blockade; understanding the basis for these outlier responses will provide potential biomarkers for therapeutic guidance and may provide insights into improving immunotherapy outcomes in patients whose cancers lack these biomarkers.

Here we report two cases of patients with biomarker-negative tumors: a NSCLC patient whose tumor had 30 mutations and was negative for PD-L1 and a MMRp CRC patient, both of whom derived durable clinical benefit from PD-1 blockade monotherapy. Each patient maintained a T-cell response to a hotspot oncogenic mutation many years after treatment initiation: BRAF N581I [5, 6] in the NSCLC patient and AKT1 E17K [7, 8] in the CRC patient. These findings suggest that neoantigens derived from oncogenic driver mutations induce endogenous T-cell responses, which may be particularly efficacious in lower mutational burden tumors owing to the likelihood that oncogenic mutations are required for tumor survival.

## Case presentations

**Fig. 1:**
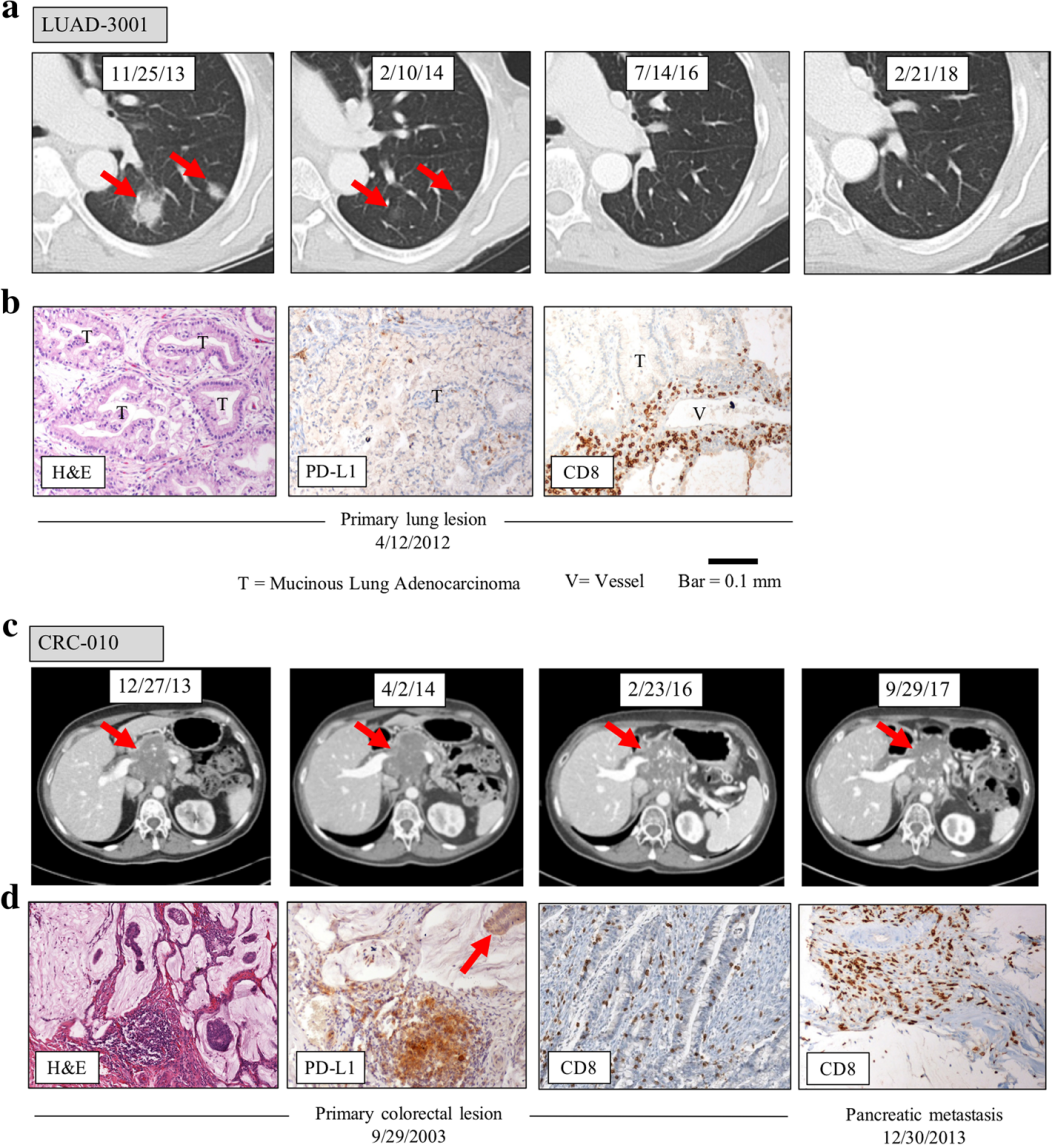
Durable clinical benefit to PD-1 blockade in two patients without high mutational burden tumors. **a**, Patient LUAD-3001 – a 76 year old woman with metastatic non-small cell lung cancer. Selected cropped IV contrast enhanced CT images of the chest in lung window at four different timepoints. Baseline exam (11/25/13) demonstrates two left lower lobe solid nodules with surrounding ground glass opacities (red arrows) compatible with metastases. First followup exam while on nivolumab (2/10/14) demonstrates near complete resolution with minimal residual ground glass opacities (red arrows). Additional two and four year followup exams (7/14/16 and 2/21/18) demonstrate complete and durable resolution of metastases, with no evidence of progression elsewhere in the body (not shown). **b**, H&E staining (left panel), PD-L1 staining (center panel), and CD8 infiltration (right panel) of the primary tumor obtained from patient LUAD-3001 during surgical resection on 4/12/2012. **c**, Patient CRC-010 - a 69 year old woman with metastatic recurrent mismatch repair proficient colorectal cancer with locally invasive pancreatic metastasis. Selected IV contrast enhanced CT images of the abdomen in venous phase. Baseline exam (12/27/13) demonstrates a heterogeneous hypovascular mass with scattered calcifications (red arrow). Four month follow up exam on pembrolizumab (4/2/14) demonstrates slight enlargement without new metastases. Metastasis slowly decreased in size on 2 year follow up (2/23/16) and slightly increased on four year follow up exam (9/29/17). No new metastases are seen on interval or latest CT exam and disease remains stable. **d**, H&E staining (left panel), PD-L1 staining showing no expression on tumor cells (red arrow, center left panel) but high expression at the invasive front on a discrete immune cell aggregate and CD8 infiltration (center right panel) in the primary tumor obtained from patient CRC-010 during surgical resection on 9/29/2003. CD8 staining demonstrating a brisk CD8^+^ lymphocytic infiltrate is also shown on a fine needle aspiration of the pancreatic recurrence on 12/30/2013 (right panel)

We evaluated two patients with tumors anticipated to be non-responsive to immunotherapy who derived durable clinical benefit and prolonged overall survival from anti-PD-1 therapy – both remain alive with ECOG-0 performance status years later. The first patient, LUAD-3001, is a 76-year-old woman who underwent a right lower lung lobectomy in 2012 for a T3 N0 well-to-moderately differentiated mucinous adenocarcinoma. Nine months after finishing adjuvant chemotherapy, three new lung nodules were found on surveillance imaging. Biopsy confirmed recurrent adenocarcinoma. The patient was enrolled on a clinical trial of single-agent nivolumab in December 2013 (NCT01454102), with therapy ongoing through the present. By July 2014, the metastases had completely disappeared, and complete response continues to be maintained for 4.5 years. Figure 1a shows LUAD-3001 CT before treatment, at first follow up, as well as 2.5 and 4 years after follow up. Whole exome sequencing revealed the patient’s tumor had 30 nonsynonymous exome mutations and was negative for ALK, EGFR, ROS1, and KRAS abnormalities. The tumor harbored an oncogenic BRAF N581I mutation [5, 6] (Additional file 1: Table S1). The tumor cells were negative for PD-L1 expression (Fig. 1b, center), though prominent perivascular lymphoid aggregates were PD-L1+. Immunophenotyping confirmed the presence of CD8^+^ T lymphocytes (Fig. 1b, right).

The second patient, CRC-010, is a 69 year old woman initially diagnosed with a stage III mucinous right-sided colon adenocarcinoma. PD-L1 expression in her original primary tumor was observed at the interface of tumor and normal tissue and there was a dense CD8^+^ lymphocytic infiltrate **(**Fig. 1d). Staining for mismatch repair enzymes was normal, consistent with a mismatch repair proficient genotype. Whole exome sequencing of the primary lesion revealed 118 mutations, including oncogenic BRAF V600E and AKT1-E17K mutations. There were no mutations in any of the genes encoding mismatch repair proteins, KRAS or NRAS (Additional file 1: Table S1Table S2).Ten years after a right hemicolectomy, FOLFOX adjuvant therapy and FOLFIRI/cetuximab, she developed a pancreatic metastatic recurrence in 2013. The patient began pembrolizumab therapy in January 2014. CT scans in April 2014 showed disease stabilization. In May 2014, she discontinued pembrolizumab after 4 doses due to grade 3 serum pancreatic enzyme elevation. CT at the time showed stable disease with no new metastases (Fig. 1c). Following a course of chemoradiation with capecitabine, and then FOLFOX/Bevacizumab, she has not received further therapy since May 2015 and imaging studies continue to show a stable pancreatic mass with no new lesions (Fig. 1c). A fine needle aspiration biopsy of the pancreatic mass performed at the end of treatment in June 2015 demonstrated the presence of clusters of neoplastic cells compatible with residual moderately differentiated adenocarcinoma with mucinous features and a brisk infiltrate of CD8^+^ T-cells (Fig. 1d).

## Methods

### Patient selection and tumor samples

The patients described in this study provided informed consent as approved by the IRB of Johns Hopkins. Patient LUAD-3001 was enrolled to CheckMate 012, a phase I study evaluating nivolumab combination therapy in subjects with stage IIIb/IV non-small cell lung cancer (NSCLC; clinicaltrials.gov, NCT01454102) and was treated with nivolumab monotherapy [9]. Patient CRC-010 was enrolled to a phase II study of pembrolizumab treatment for metastatic colorectal cancer (CRC; clinicaltrials.gov, NCT018706511) [1]. The samples used for each analysis in this study are detailed in Additional file 1: Table S1Table S3.

### Histopathology, immunohistochemistry and image analysis

Tissue specimens were stained with hematoxylin and eosin combination (H&E). Formalin-fixed paraffin embedded (FFPE) tissue sections were stained for CD8 (clone C8144B, Cell Marque, Rocklin, CA) and PD-L1 (clone E1L3N) as previously reported [10].

### Whole exome sequencing (WES), neoantigen prediction, and in vitro peptide binding assays

Tumor and normal WES were compared to identify somatic alterations using the VariantDx software pipeline [11]. Mutations from WES combined with each patient’s major histocompatibility complex class I haplotype were applied in the ImmunoSelect-R neoantigen prediction platform (Personal Genome Diagnostics) [11]. This algorithm predicts the MHC class I binding potential of each somatic and wild-type peptide. Neoantigen candidates were further filtered by tumor-associated expression levels derived from TCGA to generate a final peptide ranking for experimental testing. Lollipop plots showing mutations detected in the *BRAF* and *AKT1* genes were generated by cBioPortal [12, 13]. Binding assays were performed as previously described [14].

### Peripheral blood T-cell reactivity and bioinformatic identification of mutation associated neoantigen-specific T-cell clonotypes

We used the MANAFEST (Mutation Associated NeoAntigen Functional Expansion of Specific T-cells) assay [15] to evaluate T-cell responsiveness to mutation-associated neoantigens. Briefly, putative neoantigenic peptides defined by the ImmunoSelect-R pipeline (see above [11]; Additional file 1: Table S1Tables S4 and S5) were synthesized (Sigma-Aldrich) and used to stimulate T-cells in vitro for 10 days as previously described [15]. T-cell receptor sequencing (TCRseq; Adaptive Biotechnologies) [16] was performed on individual peptide-stimulated T-cell cultures and T-cells cultured without peptide. Bioinformatic analysis of productive clones was performed to identify antigen-specific T-cell clonotypes meeting the following criteria: 1) significant expansion (Fisher’s exact test with Benjamini-Hochberg correction for FDR, *p* < 0.0001) compared to T-cells cultured without peptide, 2) significant expansion compared to every other peptide-stimulated culture (FDR < 0.0001), 3) an odds ratio > 5 compared to the “no peptide” control, 4) a minimum of 10 templates detected by TCRseq, and 5) reached a minimum baseline threshold to ensure adequate distribution among culture wells or was detected in a repeat stimulation experiment. TCRseq was also performed on DNA extracted from tumor tissue obtained from the primary surgical resection and serial peripheral blood samples where available. TCRseq was performed using the survey resolution ImmunoSEQ® platform for tissue and MANAFEST samples and deep resolution sequencing for peripheral blood samples [16].

## Results

### T-cell recognition of mutation-associated neoantigens

**Fig. 2:**
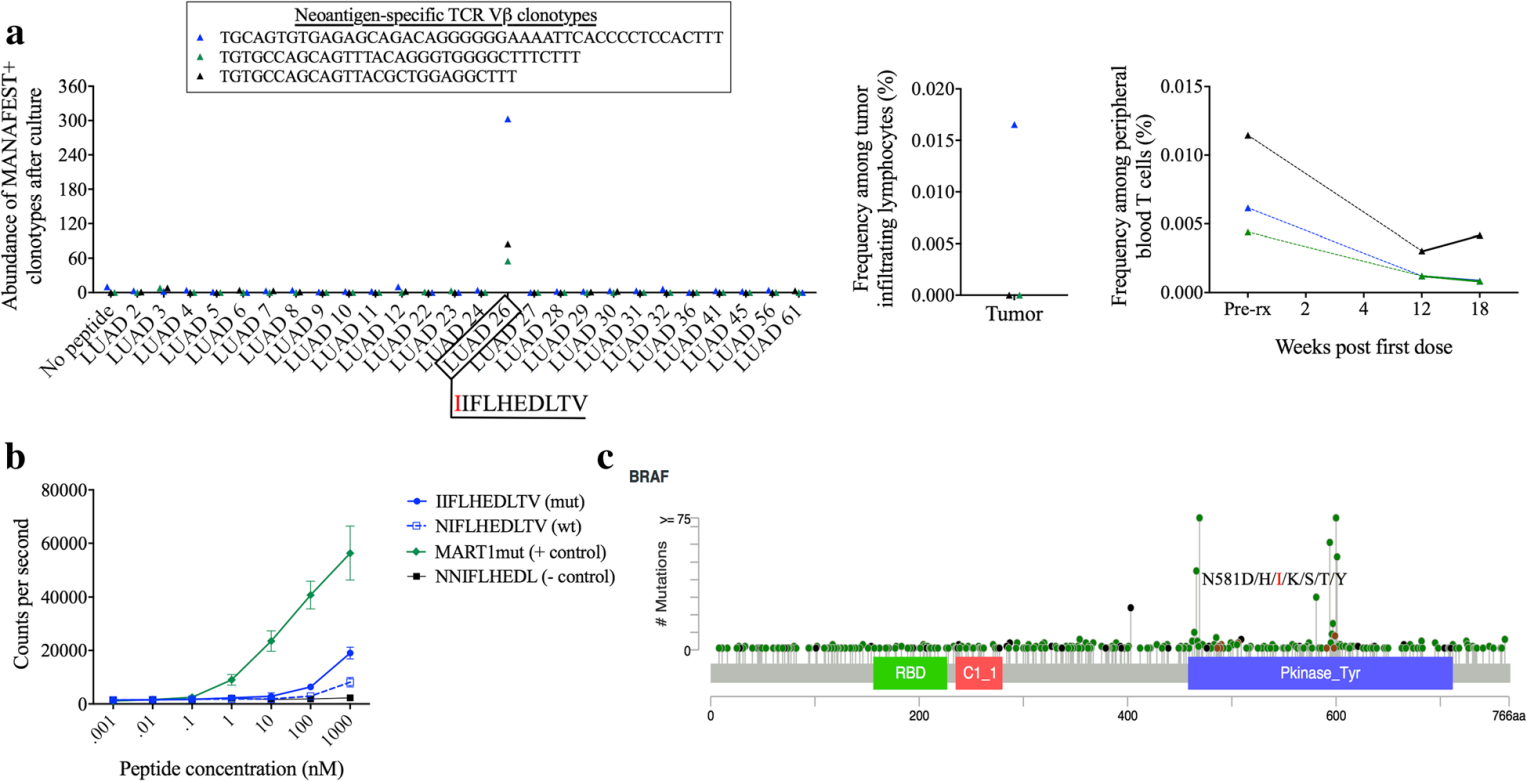
T-cell recognition of BRAF N581I mutation in lung cancer patient LUAD-3001 responding to anti-PD-1 treatment. Individual 10-day peptide-stimulated cultures identified persistent mutation associated neoantigen-specific clonotypes (described in methods) detectable in the blood of patient LUAD-3001 > 2 years after complete tumor regression following PD-1 blockade. **a** Three clonotypes recognized the A*02:01-restricted BRAF N581I-derived IIFLHEDLTV peptide neoantigen (LUAD 26, left panel). The TGCAGTGTGAGAGCAGACAGGGGGGAAAATTCACCCCTCCACTTT clonotype was detected in the original resected tumor (center panel), whereas all three clonotypes were detected in serial peripheral blood samples obtained before and after PD-1 blockade (right panel). Data are shown as the number of cells detected after the 10 day culture (abundance) for cultured cells and the relative frequency (%) of each clonotype among all cells detected by TCRseq for FFPE tumor tissue and serial peripheral blood samples. **b** Duplicate binding assays were performed on the putative neoantigen and wild type counterpart, as well as the known MART1 mutant HLA A*02:01-restricted ELAGIGILTV epitope. Data are shown as mean counts per second, with error bars representing the standard deviation. **c** The lollipop plot shows the position of the patient’s BRAF N581I mutation among the other oncogenic mutations within the BRAF gene; green: missense mutations, black: truncating mutations, brown: inframe mutations, purple: other

To determine if patients LUAD-3001 and CRC-010 had circulating T-cell clones that recognized tumor neoantigens and that were also present in the tumor, we used the MANAFEST assay [15], in which short-term cultures of peripheral blood T-cells with individual candidate mutation associated neoantigen peptides predicted by an HLA-I allele-specific algorithm are analyzed via T-cell receptor sequencing (TCRseq) [16]. Twenty three out of 26 candidate neoantigenic peptides tested induced significant and specific clonotypic expansions of CD8^+^ T cells obtained from patient LUAD-3001 2 years after anti-PD-1 treatment initiation (Additional file 1: Table S1Table S6). Two of these neoantigens (a 10mer and an 11mer; LUAD 26 and LUAD 31) contained the oncogenic driver mutation BRAF N581I, a hotspot mutation previously reported to recurrently occur in melanoma and colorectal cancer [5, 6, 17]. The oncogenic mechanism underlying BRAF N581I is distinct from that of BRAF V600E in that N581I has diminished or inactive BRAF kinase activity but induces KRAS-dependent CRAF signaling and ERK activation [17]. Three T-cell clones reactive with BRAF N581I recognized LUAD 26, a HLA-A*02:01-restricted epitope (IIFLHEDLTV; Fig. 2a, Additional file 1: Table S1Table S6); one of these clones was detected in the original primary tumor resection. All three of these clones were detected in peripheral blood T cells obtained prior to treatment, and were present at much lower frequency by 12 weeks after treatment initiation and after complete tumor regression. Although having seemingly poor binding affinity for HLA-A*02:01, LUAD 26 demonstrated improved binding kinetics relative to its wild-type counterpart (Fig. 2c). T cell recognition of hotspot BRAF mutations have previously been described [18, 19], but this is the first report of a T cell response against neoantigens derived from mutations at position 581. Remarkably, T-cell recognition of the BRAF N581I oncogenic driver and 22 additional mutation-associated neoantigens described herein (Additional file 1: Table S1Table S6) persisted years after complete tumor regression, thereby defining a preexisting and long-lived antitumor memory T-cell response.

**Fig. 3:**
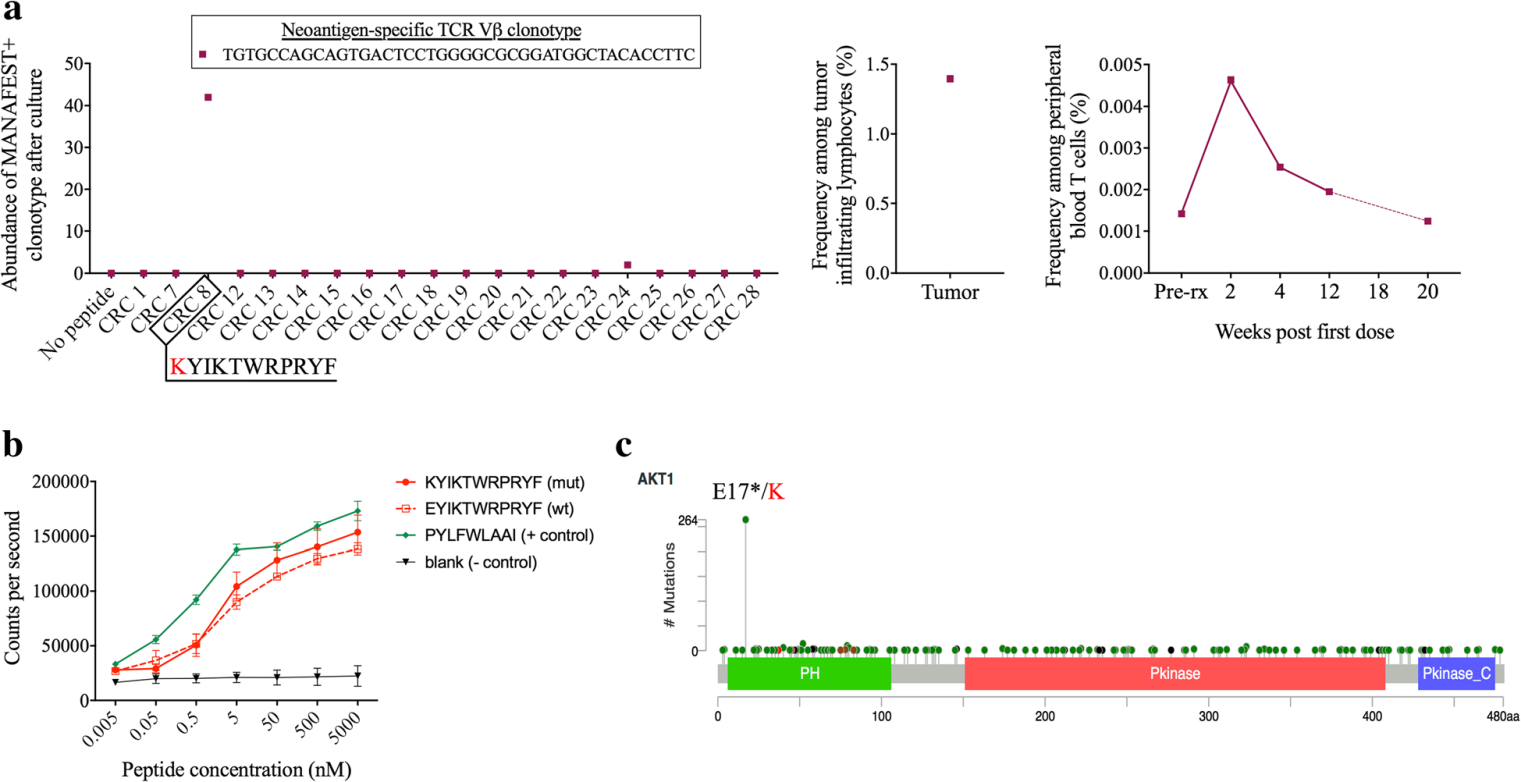
T-cell recognition of AKT1 E17K mutation in MMRp CRC-010 with stable disease after anti-PD-1 treatment. Individual 10-day peptide-stimulated cultures identified long-lived mutation associated neoantigen-specific clonotypes (described in methods) detectable in the blood of patient CRC-010 3 years after developing stable disease following PD-1 blockade: **a** The TGTGCCAGCAGTGACTCCTGGGGCGCGGATGGCTACACCTTC clonotype, which recognized the HLA-A*23:01-restricted AKT1 E17K-derived KYIKTWRPRYF peptide neoantigen (CRC8, left panel), was detected in the original resected tumor (center panel) and expanded in the periphery upon pembrolizumab treatment (right panel). Data are shown as the number of cells detected after the 10 day culture (abundance) for cultured cells and the relative frequency (%) of each clonotype among all cells detected by TCRseq for FFPE tumor tissue and serial peripheral blood samples. **b** Duplicate binding assays were performed on the putative neoantigen and wild type counterpart, as well as the known HLA A*23:01-restricted EBV PYLFWLAAI epitope as a positive control. Data are shown as mean counts per second, with error bars representing the standard deviation. **c** The lollipop plot shows the position of the patient’s AKT1 E17K mutation among the other oncogenic mutations within the AKT1 gene; green: missense mutations, black: truncating mutations, brown: inframe mutations, purple: other

Similarly to patient LUAD-3001, we detected T cell reactivity to the oncogenic driver mutation, AKT1 E17K, in peripheral T cells obtained 3 years post-anti-PD-1 from patient CRC-010, a patient with MMRp mCRC. We identified two specifically recognized mutation-associated neoantigen peptides (Additional file 1: Table S1Table S6), including an AKT1 E17K – derived, HLA-A*23:01-restricted KYIKTWRPRYF peptide epitope (CRC 8) that induced a single expanded TCRVβ clonotype (Fig. 3a). This clone persisted in the periphery of the tumor-bearing patient as evidenced by its detection in a a subsequent blood sample collected ~one year later (data not shown). Strikingly, this single T-cell clone comprised 1.4% of tumor infiltrating lymphocytes detected in the original primary colon tumor of patient CRC-010 and underwent rapid expansion in the periphery upon PD-1 blockade before returning to pre-treatment frequency by 20 weeks post-treatment (Fig. 3a). This neoantigen demonstrated high affinity binding to A*23:01 in an in vitro assay, with similar binding kinetics observed in the wild-type peptide (Fig. 3b). The E17K mutation is a “hotspot” in AKT1, accounting for the majority of mutations causing constitutive activation of the kinase [8].

## Discussion and conclusions

These findings demonstrate that driver mutations may elicite efficient long-lived endogenous anti-tumor immune responses, and these responses may facilitate clinical response in patients treated with checkpoint blockade. On this note, adoptive transfer of T cells specific for hotspot driver oncogenic mutations, including CD8^+^ T cells specific for an HLA class I-restricted KRAS G12D epitope [20] and CD4^+^ T cells specific for an HLA class II-restricted BRAF V599E [19] or BRAF V600E [18] mutation have proven to derive clinical benefit. Here we provide further demonstration that endogenous memory T-cells targeting such oncogenic driver mutations can persist in the peripheral blood for many years after tumor clearance. Interestingly, the BRAF N581I-derived neoantigen demonstrated limited affinity for HLA-A*02:01 in our in vitro binding assay (Fig. 2b). Lower affinity epitopes (> 500 nM) are not uncommon [21–23] and in some instances binding to HLA might be subsequently enhanced by post-translational modifications [24]. This is in contrast to the high affinity of the AKT1 E17K-derived neoantigen and its wild-type counterpart. In this case,the mutated amino acid is located at position 1 and therefore unlikely to affect binding to the MHC but may interfere with cognate TCR binding.

The identification of immunogenic oncogene-derived neoantigens has profound clinical implications. In contrast to passenger mutations, driver oncogenic mutations are less likely to be eliminated by the tumor as a means of immune escape, since they are required for the transformed phenotype. Thus, sustained T cell responses against driver mutations likely are more productive for long term tumor control [25]. New bioassays to detect and monitor the immune response to neoantigens, such as the MANAFEST assay used here, will help systematic screening for T-cell responses against tumor-specific mutations with a special emphasis on oncogenic driver mutations. In addition, the development of new assays that enable concurrent phenotypic profiling of neoantigen-specific T cell clonotypes will shed light on the effector function of these T cells. They may delineate a patient population that would not otherwise be predicted to respond to checkpoint blockade based on current biomarkers, such as tumor PD-L1 expression, high TMB, or mismatch repair status. While these findings do not provide evidence that the oncogene reactive T cells facilitated durable clinical benefit in these patients, they provide the foundation for further exploration of biomarkers likely to identify previously unappreciated populations of patients eligible for checkpoint blockade-based clinical trials. Indeed, patient LUAD-3001 would not currently be eligible to receive anti-PD-1 monotherapy as standard of care in the first line setting, as she did as part of CHECKMATE 012, and patient CRC-010 would not be eligible to receive anti-PD-1 at all. Additionally, identifying T cell clonotypes specific for these mutations provides the foundation for vaccines or T cell therapies targeting oncogene mutation-derived neoantigens in patients who did not mount these responses endogenously. High TMB, tumor PD-L1 expression, and MMR status being imperfect predictive biomarkers, new sensitive next generation sequencing methods, T-cell detection assays, and epitope prediction algorithms allow for the systematic screening of cancer patients for reactivity towards shared driver mutations, the detection of which could provide additional predictive value for clinical benefit to checkpoint blockade.

## Additional file

Additional file 1: **Table S1.** Sequence alterations detected in the primary tumor of LUAD-3001, **Table S2.** Sequence alterations detected in the primary tumor of CRC-010, **Table S3.** Specimen characteristics used for genomic and immunologic analyses, **Table S4.** Putative neoantigens tested in LUAD-3001, **Table S5.** Putative neoantigens tested in CRC-010, **Table S6.** Neoantigen-specific T cell clonotypes detected in patients LUAD-3001 and CRC-010 (XLSX 98 kb)

## Abbreviations

NSCLC: Non-small cell lung cancer
CRC: Colorectal cancer
PD-1: Programmed death 1
PD-L1: Programmed death ligand 1
TMB: Tumor mutational burden
MMRd: Mismatch repair deficient
MSI-H: Microsatellite instabibility high
MMRp: Mismatch repair proficient
MSS: Microsatellite stable
ECOG: Eastern Cooperative Oncology Group
FFPE: Formalin fixed, paraffin embedded
CD8: Cluster of differentiation 8
WES: Whole exome sequencing
TCGA: The Cancer Genome Atlas
MANAFEST: Mutation associated neoantigen functional expansion of specific T cells
TCRseq: T cell receptor sequencing
DNA: Deoxyribonucleic acid
HLA: Human leukocyte antigen
TCRVβ: T cell receptor variable gene, β chain
MHC: Major histocompatibility complex
