# Correction to: persistent mutant oncogene specific T cells in two patients benefitting from anti-PD-1

**DOI:** 10.1186/s40425-019-0547-7

**Published:** 2019-03-06

**Authors:** Kellie N. Smith, Nicolas J. Llosa, Tricia R. Cottrell, Nicholas Siegel, Hongni Fan, Prerna Suri, Hok Yee Chan, Haidan Guo, Teniola Oke, Anas H. Awan, Franco Verde, Ludmila Danilova, Valsamo Anagnostou, Ada J. Tam, Brandon S. Luber, Bjarne R. Bartlett, Laveet K. Aulakh, John-William Sidhom, Qingfeng Zhu, Cynthia L. Sears, Leslie Cope, William H. Sharfman, Elizabeth D. Thompson, Joanne Riemer, Kristen A. Marrone, Jarushka Naidoo, Victor E. Velculescu, Patrick M. Forde, Bert Vogelstein, Kenneth W. Kinzler, Nickolas Papadopoulos, Jennifer N. Durham, Hao Wang, Dung T. Le, Sune Justesen, Janis M. Taube, Luis A. Diaz Jr, Julie R. Brahmer, Drew M. Pardoll, Robert A. Anders, Franck Housseau

**Affiliations:** 1 Bloomberg-Kimmel Institute for Cancer Immunotherapy, Johns Hopkins University, Baltimore, MD USA; 2 Sidney Kimmel Comprehensive Cancer Center, Johns Hopkins University, Baltimore, MD USA; 3 Department of Pathology, Johns Hopkins University, Baltimore, MD USA; 4 Russell H. Morgan Department of Radiology and Radiological Science, Johns Hopkins University, Baltimore, MD USA; 5 Division of Biostatistics and Bioinformatics, Johns Hopkins University, Baltimore, MD USA; 6 The Swim Across America Laboratory, John Hopkins University, Baltimore, MD USA; 7 Ludwig Center and Howard Hughes Medical Institute, Johns Hopkins University, Baltimore, MD USA; 8 Immunitrack, Copenhagen, Denmark; 9 Department of Medicine, Division of Solid Tumor Oncology, Memorial Sloan Kettering Cancer Center, New York, NY USA; 10 Present address: B.R.B., Bioinformatics Core, Department of Complementary & Integrative Medicine, University of Hawaii John A. Burns School of Medicine, Honolulu, HI 96813 USA

**Correction to: Journal for ImmunoTherapy of Cancer 2019 7:40**

10.1186/s40425-018-0492-x

Following publication of the original article [1], it was reported that not all authors’ competing interests were stated. The updated Competing Interests can be seen below.

**Competing interests**

K.N.S., F.H., D.M.P., V.A., V.E.V., B.V., K.W.K., N.P. and L.A.D. have filed for patent protection on a subset of the technologies described herein (US provisional application no. 62/407,820).

D.M.P. has ownership interest (including patents) in BMS, MedImmune/AstraZeneca, and Potenza, and is a consultant/advisory board member for BMS and MedImmune/AstraZeneca.

V.E.V. is a founder of, holds equity in, and is a member of the Board of Directors of Personal Genome Diagnostics (PGDx). Under license agreements between PGDx, as well other companies, and the Johns Hopkins University, V.E.V. is entitled to a share of royalties received by the University on sales of services or products described in this paper. Within the last five years, V.E.V. has been an advisor to Daiichi Sankyo, Janssen Diagnostics, Ignyta, and Takeda Pharmaceuticals. These arrangements have been reviewed and approved by the Johns Hopkins University in accordance with its conflict of interest policies.

B.V., K.W.K., & N.P. are members of the Scientific Advisory Board of Sysmex and are founders of PapGene and Personal Genome Diagnostics and advise Sysmex. KWK & BV advise Eisai, Phoremost, and Morphotek, and BV is also an advisor to Camden Partners. The companies named above, as well as other companies have licensed previously described technologies related to the work described in this paper from Johns Hopkins University. Some of these licenses are or will be associated with equity or royalty payments to BV, KWK, NP, and IK. Additional patent applications on the work described in this paper may be filed by Johns Hopkins University. The terms of all these arrangements are being managed by Johns Hopkins University in accordance with its conflict of interest policies.

D.T.L. serves on advisory boards for Merck and Bristol Myers Squibb and has received research funding from Merck, Bristol Myers Squibb, Aduro Biotech, Curegenix, and Medivir. She has received speaking honoraria from Merck and is an inventor of licensed intellectual property related to technology for mismatch repair deficiency for diagnosis and therapy (WO2016077553A1) from Johns Hopkins University. The terms of these arrangements are being managed by Johns Hopkins.

L.A.D. is a member of the board of directors of Personal Genome Diagnostics (PGDx) and Jounce Therapeutics. LAD holds equity in PapGene, Personal Genome Diagnostics (PGDx) and Phoremost. He is a paid consultant for Merck, PGDx and Phoremost. LAD is an inventor of licensed intellectual property related to technology for circulating tumor DNA analyses and mismatch repair deficiency for diagnosis and therapy (WO2016077553A1) from Johns Hopkins University. These licenses and relationships are associated with equity or royalty payments to LAD. The terms of all these arrangements are being managed by Johns Hopkins and Memorial Sloan Kettering in accordance with their conflict of interest policies. In addition, in the past 5 years, LAD has participated as a paid consultant for one-time engagements with Caris, Lyndra, Genocea Biosciences, Illumina and Cell Design Labs.

J.R.B. is a consultant/advisory board member of BMS, Merck, Medimmune/AstraZeneca, Johnson and Johnson, Syndax, Celgene, Amgen, EliLilly. JB received grant funding from BMS.

P.M.F. is a consultant/advisory board member of Abbvie, AstraZeneca, Bristol-Myers Squibb, Boehringer lngelheim, EMD Serono, lnivata, Janssen, Lilly, Merck, Novartis. PF received grant/research Support from AstraZeneca, Bristol-Meyers Squibb, Corvus, Kyowa, Novartis.

J.N. is a consultant/advisory board member for Bristol Myers Squibb, MedImmune/AstraZeneca, and Roche/Genentech, has received research funding from Merck and MedImmune/AstraZeneca, and has received honoraria from Bristol Myers Squibb and MedImmune/AstraZeneca.

J.M.T. is consultant/advisory board member for Bristol-Myers Squibb, Astra Zeneca, and Merck. J.M.T. receives research funding from Bristol-Myers Squibb.

R.A.A. is a consultant/advisory board member for Bristol Myers Squibb, Merck, AstraZeneca, Adaptive Biotechnologies and has received research funding from Bristol Myers Squibb, Stand-up to Cancer and contractual work from Five Prime Therapeutics and FLX Bio.

C.L.S., F.H., N.J.L. receive research grant support from Bristol-Myers Squibb. N.J.L.: Master Nonclinical Research Agreement between Johns Hopkins University and Bristol-Myers Squibb Company; Pediatric Advisory Council BMS.
